# Effectiveness and safety of fluoxetine for premature ejaculation: Protocol for a systematic review

**DOI:** 10.1097/MD.0000000000014481

**Published:** 2019-02-15

**Authors:** Xiao Li, Juntao Li, Xianrui Li, Jisheng Wang, Hengheng Dai, Jingshang Wang

**Affiliations:** a Department of Andrology, Dongzhimen Hospital, Beijing; b Department of Andrology, The First Affiliated Hospital of Henan University of Chinese Medicine, Zhengzhou; c Department of Traditional Chinese Medicine, Beijing Obstetrics and Gynecology Hospital, Capital Medical University, Beijing, China.

**Keywords:** fluoxetine, premature ejaculation, protocol, systematic review

## Abstract

**Background:**

Premature ejaculation (PE) is one of the most common male sexual dysfunctions, which can directly harm men's self-esteem and affect the stability of the relationship between husband and wife. To some extent, PE even affects the harmony and stability of society. So, men's health has gained more and more attention. As one of the long-acting selective serotonin reuptake inhibitors (SSRIs), fluoxetine has been proven to be effective in the treatment of PE by many trails. In this study, we aim to evaluate the effectiveness and safety of fluoxetine for PE to provide the newest evidence for clinical use.

**Methods and analysis:**

Literature research will be divided into 2 parts: electronic search and manual search. We will search PubMed, EMBASE, The Cochrane Library, the China National Knowledge Infrastructure (CNKI), China Biology Medicine disc (CBMdisc), the China Science and Technology Journal database (VIP), and the Wanfang database online. We will select the eligible studies published up to December 31, 2018. Manual searches mainly retrieve dissertations, ongoing trails, internal reports, and so on. We use intravaginal ejaculatory latency time (IELT) as the primary outcome of PE and we also care about the following indexes: PE Diagnostic Tool (PEDT); Arabic index of PE (AIPE); Index of PE (IPE). In addition, we will carefully observe the patient's adverse reactions during the medication. Two reviewers will read the articles, extract the data information, and assess the risk of bias independently. Data analysis will be used the software such as RevMan V.5.3.5; EndNote X7 and Stata 13.0.

**Results:**

This study will provide a high-quality synthesis of current evidence of fluoxetine for PE from several aspects, including IELT, PEDT, AIPE, IPE, and adverse events.

**Conclusion:**

This systematic review will provide evidence to assess the effectiveness and safety of fluoxetine in the treatment of PE.

**Trial registration number:**

PROSPERO CRD42018109722.

## 1 Introduction

Premature ejaculation (PE) is one of the most common male sexual dysfunctions; about 21% to 33% of male populations are troubled by PE.^[1,2]^ PE can directly harm men's self-esteem and affect the stability of the relationship between husband and wife. To some extent, PE even affects the harmony and stability of society.^[3,4]^ So, men's health has gained more and more attention.

Currently, we do not fully understand the cause of PE, and there is no consensus on the definition of PE either. Waldinger et al^[5,6]^ used intravaginal ejaculation latency time (IELT) to define PE, which averaged 5.4 minutes (0.55–44.10 min) from initial insertion to ejaculation, and defined IELT < 1 minute as PE, and 1.0 to 1.5 minutes is considered abnormal. At the same time, they aim that there are 4 PE subtypes: lifelong PE (LPE), acquired PE (APE), variable PE, and subjective PE.^[7,8]^ As the research progresses, the definition of PE is constantly updated by the International society for sexual medicine (ISSM). The definitions now include: IELT often less than 1 minute (LPE) or less than 3 minutes (APE); decreased ejaculation control ability; and negative emotions (such as anxiety, depression, pessimism, etc).^[9]^ At present, IELT is commonly used in clinical diagnosis and evaluation of PE treatment effects. In addition, PE Diagnostic Tool (PEDT),^[10]^ Arabic index of PE (AIPE),^[11]^ and Index of PE (IPE)^[12]^ are also used widely.

At present, treatments for PE mainly include psychological and behavioral therapy,^[13]^ oral drug therapy, and local drug anesthesia therapy.^[14]^ Oral selective serotonin reuptake inhibitors (SSRIs) have become the most common method for treating PE because they are effective and easy to use, including fluoxetine, paroxetine,^[15]^ dapoxetine,^[16]^ sertraline,^[17]^ and so on. As one of the long-acting SSRIs, fluoxetine has been proven to be effective in the treatment of PE in many trials.^[18–21]^ However, its efficacy remains to be further evaluated. Besides, the publication of the similar systematic review has not been retrieved in the database. Therefore, this review hopes evaluate the effectiveness and safety of fluoxetine in the treatment of PE to provide the newest evidence for clinical use.

## 2 Methods

The protocol has been registered on PROSPERO as CRD42018109722. (https://www.crd.york.ac.uk/prospero/display_record.php?RecordID=109722). The protocol follows the Preferred Reporting Items for Systematic Reviews and Meta-Analyses Protocols (PRISMA-P) statement guidelines. We will describe the changes in the full review if necessary.

### 2.1 Inclusion criteria for study selection

#### 2.1.1 Types of studies

Randomized controlled trials (RCTs) with language limited to Chinese and English of fluoxetine for the treatment of PE will be included in the research, regardless of whether blinding or allocation concealment was adopted. Non-RCTs, series of case reports, and cross-research will be excluded.

#### 2.1.2 Types of participants

The cases included are adult male patients over 18 years of age who have been diagnosed with PE. Meanwhile, there is no limitation for the region, nation, ethnic, and sources.

#### 2.1.3 Types of interventions

The treatment group will use the fluoxetine, with no limit of the dose and frequency of the medicine. At the same time, the control group will use placebo for intervention.

#### 2.1.4 Types of outcome measures

##### 2.1.4.1 Primary outcomes

We use IELT as the primary outcome of PE.

##### 2.1.4.2 Secondary outcomes

We also care about the following indexes: PEDT; AIPE; IPE. In addition, we will carefully observe the patient's adverse reactions during the medication.

### 2.2 Search methods for the identification of studies.

#### 2.2.1 Electronic searches

Literature research will be divided into 2 parts: electronic search and manual search. We will search PubMed, EMBASE, The Cochrane Library, the China National Knowledge Infrastructure (CNKI), China Biology Medicine disc (CBMdisc), the China Science and Technology Journal database (VIP), and the Wanfang database online. We will select the eligible studies published up to December 31, 2018. The search terms used in the systematic review are as follows: fluoxetine, selective serotonin reuptake inhibitors, SSRIs, premature ejaculation, and PE.

The specific search strategy will be (taking PubMed as an example):

Search ((((Premature ejaculation[Title/Abstract] OR PE[Title/Abstract] OR sexual dysfunction [Title/Abstract]))) AND (Fluoxetine[Title/Abstract] OR Selective Serotonin Reuptake Inhibitors [Title/Abstract] OR SSRIs [Title/Abstract]))

And similar search strategy will be applied to other electronic databases.

#### 2.2.2 Searching other resources

Manual searches mainly retrieve dissertations, ongoing trails, internal reports, and so on to avoid missing any relevant studies. At the same time, we will also retrieve ongoing trials related to PE from the clinical registration platform, such as the WHO International Clinical Trial Registry Platform (ICTRP). And we will try to acquire complete information if needed.

### 2.3 Data collection and analysis

#### 2.3.1 Selection of studies

**Figure 1:**
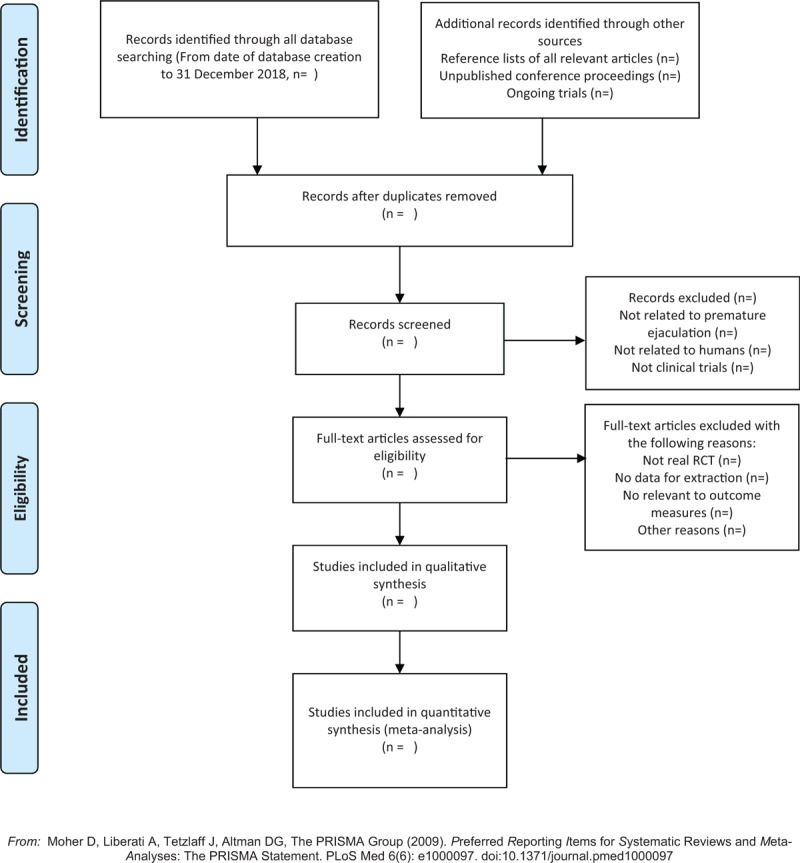
The PRISMA flow chart.

Two reviewers will independently read the titles of the references first to exclude the literature that are obviously irrelevant, and then read the abstract and full text to determine whether the studies would be finally included. The screening process will use EndNote X7 (Thomson Reuters Corporation, Stanford, America) literature management software. As for the incomplete information, the reviewers shall contact the author for the complete information. If there is a disagreement, it will be decided with another reviewer. The exclusion reason for the excluded literature will be recorded. The details of selection process will be shown in the PRISMA flow chart (Fig. 1).

#### 2.3.2 Data extraction and management

Two reviewers will retrieve the following data independently according to the data collection table designed beforehand. The following information will be extracted: study details (authors, country, year of publication, multicenter study or not), participant details (baseline data, diagnostic criteria of PE), the methods used (sample size, blinding method), the interventions used in both treatment and control group, the primary and secondary outcomes (IELT, PEDT, AIPE, IPE, adverse events). We will contact the corresponding authors for the data mentioned above if the data are incomplete. And all reviewers will join the discussion to solve the disagreement.

#### 2.3.3 Assessment of risk of bias in included studies

Two reviewers will assess the quality of each article independently using the risk of bias (ROB) assessment tool in the Cochrane. Evaluation criteria include random sequence generation, allocation concealment, blindness assessment of results, incomplete outcome data, selective reporting, and other biases. The evaluation results will be divided into 3 levels: low-risk, unclear, and high-risk. If there is a disagreement, we will conduct group discussions or discuss with another reviewer to make a decision.

#### 2.3.4 Measures of treatment effect

We will record the mean difference (MD) or standardized mean difference (SMD) and 95% confidence interval (95% CI) for continuous variable outcomes. While for dichotomous outcomes, we will record the relative risk (RR) and 95% CI.

#### 2.3.5 Dealing with missing data

If the data we need are unclear or not reported in a clinical article, the reviewer will contact the author by phone, email, or mail to collect the missing data in the data collection form as completely as possible.

#### 2.3.6 Assessment of heterogeneity

All the literatures included will use *I*^2^ to determine the heterogeneity. When *I*^2^ ≤ 50%, heterogeneity is considered acceptable. The heterogeneity among the trials will be considered significant when *I*^2^ > 50%, and further subgroup analysis should be conducted to identify possible causes.

#### 2.3.7 Data synthesis and analysis

RevMan V.5.3.5 (The Cochrane Collaboration, Oxford, England) will be used for data synthesis and analysis. When the heterogeneity is not obvious, a fixed-effects model will be used to calculate the RR and MD; on the contrary, we will use a random-effects model to synthesize the data. If the heterogeneity is significant, we will analyze the cause of the heterogeneity and subgroup analysis or meta regression analysis will be performed.

#### 2.3.8 Assessment of publication bias

The funnel plot will be used to judge whether a publication bias exists if at least 10 trials are included in the research. And the publication bias assessment will be determined by Begg test and Egger test using STATA 13.0 (Stata version 13.0; StataCorp LP, College Station, TX) when the number is less than 10.

#### 2.3.9 Subgroup analysis

When the meta-analysis suggests significant heterogeneity, we will perform a subgroup analysis based on the type of PE and different methods of taking fluoxetine.

#### 2.3.10 Sensitivity analysis

Sensitivity analysis will be performed to examine the robustness of conclusions. We will reanalyze whether the conclusions have changed by changing the type of study (including or excluding a particular study), imputing missing data, and so on.

#### 2.3.11 Grading the quality of evidence

We will use the GRADE software to assess the quality of the included studies. Bias risks; heterogeneity; publication bias; and other factors will be considered. The quality of the evidence will be graded using “very low,” “low,” “moderate,” or “high.”

## 3 Discussion

The ejaculation activity is mainly transmitted through the spinal nerve, the motor central nervous system, etc. The serotonin system plays an important role in ejaculation control systems, providing a new way for clinical treatment of PE.^[22,23]^

**Figure 2:**
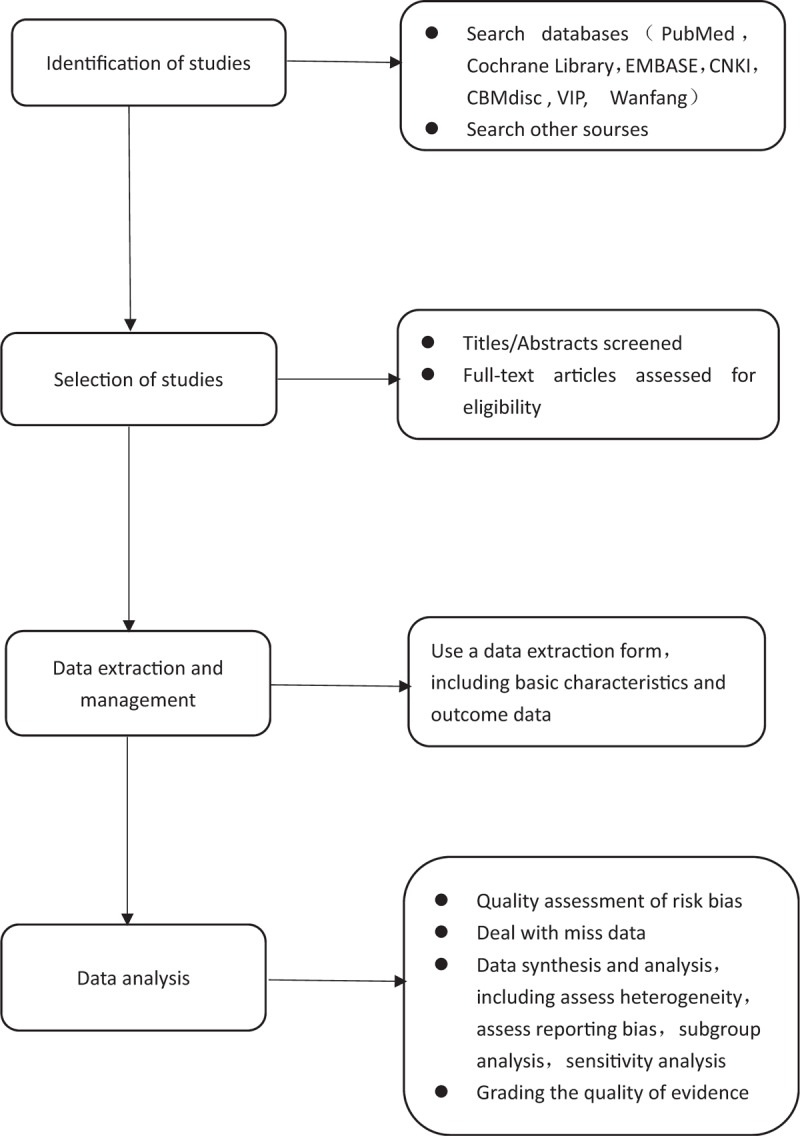
Flow diagram of the systematic review and meta-analysis.

As a long-acting SSRI, fluoxetine can effectively inhibit the reuptake of serotonin and increase the concentration of serotonin in the central nervous system to improve PE symptoms.^[24]^ In addition, Mousavizadeh et al^[25]^ demonstrated through experimental studies that fluoxetine has a blocking effect on calcium channels in tissues such as vas deferens and thus inhibits contraction of the vas deferens. Seo et al^[26]^ believe that fluoxetine can counteract the contraction of the vas deferens caused by adrenaline, thereby delaying ejaculation. At present, there are many reports that fluoxetine has a significant curative effect for PE. We hope to systematically evaluate the published RCTs of fluoxetine for PE to provide evidence for its clinical use. The process of performing this systematic review, shown in Fig. 2, including identification of studies, selection of studies, data extraction and management, and data analysis.

Nevertheless, this review still has some limitations. First, there may be a certain ROB as language limited by Chinese and English. Second, due to the different doses of fluoxetine, heterogeneity may be significant. Finally, experiments with smaller sample sizes will result in higher risk bias.

## Author contributions

**Data curation:** Xiao Li, Juntao Li.

**Formal analysis:** Xiao Li, Xianrui Li.

**Funding acquisition:** Jingshang Wang.

**Methodology:** Xianrui Li.

**Project administration:** Jisheng Wang, Hengheng Dai.

**Software:** Jisheng Wang, Hengheng Dai.

**Supervision:** Jingshang Wang.

**Validation:** Jingshang Wang.

**Visualization:** Juntao Li, Jingshang Wang.

**Writing – original draft:** Xiao Li, Jingshang Wang.

**Writing – review & editing:** Xiao Li, Jingshang Wang.
